# Supplementary material for: Are carbon emissions trading and green financial instruments synergistic? -Comprehensive quantitative research based on content analysis
Source: PLoS One. 2024 Mar 7;19(3):e0298601. doi: 10.1371/journal.pone.0298601 (PMC10919601; doi:10.1371/journal.pone.0298601)
Supplement: S1 Data — (ZIP) [file pone.0298601.s001.zip › Data/地级市Z_F工作报告.zip!/║■▒▒/╨ó╕╨╩╨/└┤╫╘╣1⁄2╓┌║┼í╛┬φ┐╦╩2╛▌═°í┐_2018.docx]

各位代表：

现在，我代表市人民政府向大会报告工作，请予审查，并请各位政协委员和列席人员提出意见。

一、2017年工作回顾

2017年，是本届政府的开局之年，党的十九大和省第十一次党代会为发展定向导航。站在新的起点，面对多重考验，我们以习近平新时代中国特色社会主义思想为指导，在省委、省政府和市委坚强领导下，紧紧依靠全市人民，聚焦发展，合力攻坚，各项工作在困难交织中稳步前行，主要指标在调整优化中逐步上行，为长远发展奠定了较好基础。一年来，我们主要做了以下工作。

（一）突出“两资一促”[1]稳增长，发展态势缓中趋稳。这一年，经济下行压力比预计的大，困难比预想的多，结果比预料的好，主要指标总量保持全省4—6位，增速稳在合理区间，质效不断提升。

坚持抓投资、强基础。配套出台28条政策[2]，签约引资1200亿元，增长30%；引进亿元项目191个，增长1.7倍。争取上级政策资金100多亿元。孝南金凤凰纸业、云梦中一科技、安陆禾丰、市高新区杭州友成等一批产业项目进展迅速；麻竹高速大悟段基本建成，汉十高铁、童家湖大桥等一批路网通道加快建设；地下综合管廊、海绵城市等一批市政工程加快推进；“一江三河”防洪保安工程全面兴建，18座水库除险加固、4条中小河流治理、64处安全饮水等一批水利项目全面完成；孝感市民之家、市文化中心等一批民生项目基本完工。扩投资发挥了稳增长“压舱石”作用。

坚持抓产业、壮实体。研究出台盐化工、建筑、物流、农旅等产业加快发展的措施，相继实施10多项惠企政策，支持企业转型、产业升级，新增规模以上工业企业50家、限额以上商贸单位135家、出口实绩企业45家，电商企业达1716家；孝感市获评全国建筑劳务输出示范基地，建筑业增加值达88亿元。建设高标准农田56.6万亩，土地流转率38.2%，粮食总产43亿斤、增产1.7亿斤，新增农民专业合作社660家、家庭农场329家，培育水产养殖示范基地31个，农机化率73.5%。应城国家矿山公园四季温泉馆、孝南卓尔小镇、安陆伍欣甸园、大悟金岭村、孝昌西域风情园等一批景点开园营业，全市接待游客2391万人次、旅游综合收入151亿元，分别增长13%和15%。企业成长、产业壮大，成为稳增长的主要支撑。

坚持抓本级、促县域。市高新区23家日资企业销售收入实现翻番，军民融合产业基地产值达180亿元；市临空区“四纵六横”骨干路网基本成形；双峰山景区改造顺利通过4A复检，重新开园。孝南3家企业和6个乡镇财税收入过亿元，汉川跻身全国投资潜力百强县，应城品种盐产能全国第一，云梦获批全国农业标准化示范县，安陆神丹入选上市“金种子”计划，大悟获评全国电子商务进农村试点县，孝昌8家企业挤入IPO绿色通道培养计划。全市4个乡镇入选全国综合实力千强镇[3]。县域经济活力增强、实力提升，成为稳增长的重要力量。

（二）突出城乡统筹优环境，发展平台逐步夯实。这一年，城市功能不断提升，发展空间不断拓展，绿色理念渐入人心。

注重立规矩、强建管、理秩序。严格执行《孝感市城乡规划条例》，市直八部门联名通告、联合执法、全域控违，拆除违法建筑1万多平方米。调整完善土地利用总规，创新基础设施建设体制，理顺市区建设事权，统一孝感城区土地市场，统一房屋征收与补偿标准，打通 “断头路”4条[4]，改造升级背街小巷40条，维修破损路面7400多平方米；垃圾处理场新区投入使用，改造集贸市场11个、公厕10座，清疏、维修管网8600米，处置闲置土地1240多亩。系统研究旧城改造历史遗留问题，形成专项工作方案，主城区47个遗留问题分批化解，惠及1.58万户4.7万人。城区禁鞭深得民心，城管执法和体制改革领先全省，公共秩序综合整治年活动持续深入，“九大举措”[5]落地见效，“六大秩序”整治[6]广泛开展，市容市貌、公共秩序明显改观。

注重抓创建、促统筹、优平台。“美丽孝感·三项创建”[7]深入推进，孝感实现省级文明城市“六连冠”，进入省级森林城市创建名录。安陆获批国家园林城市，云梦、大悟、孝昌获批省级园林城市，4个县市再次荣获省级文明城市。入选国家首批运动休闲特色小镇2个，省级特色小镇2个[8]。植树造林16.85万亩，建设美丽乡村151个。高位推进汉孝一体、市区一体、孝云一体，区域融合步伐加快。

注重建机制、重治理、严惩处。用好绿色指挥棒，强力实施扬尘治理、秸秆禁烧，淘汰黄标车2889辆、燃煤锅炉424台，空气优良天数比例达76.2%；澴东“三湖”水体修复、城区黑臭水体治理全面铺开，8个省级工业聚集区污水处理设施加快建设，89座乡镇污水处理厂全部开工，四级河（湖、库）长体系搭建完成，湖库拆围4.64万亩，关闭、搬迁禁养区养殖场583家，8个国考断面水质全部达标；测土配方施肥360多万亩次，化肥、农药用量减少近万吨。万元GDP能耗、亿元GDP地耗分别下降3.6%和6.03%，节能减排任务超额完成。

（三）突出改革创新解难题，发展制约得到缓解。这一年，坚持市场配置资源的决定性作用，更好发挥政府作用，打造优质政务环境和公平营商环境，创新创业活力增强。

市场化改革逐步深化。国企改革、“三供一业”分离移交、市本级国有资产划转有序推进，组建了湖北澴川国投和众益公交集团，公交公营、押运公司改革全面完成，盐业体制改革平稳有序。“营改增”减税18.3亿元，22家企业直供电降成本6700多万元，孝感城区商品房库存保持在合理区间，5家银行创新金融产品6个，续贷周转金、融资担保、“集合贷”“增信贷”为310家企业融资15.4亿元。农业供给侧结构性改革25个重大项目扎实推进，农村承包地确权登记颁证试点全面完成，不动产统一登记形成“孝感模式”。新型城镇化试点迎接国家评估验收，社会信用体系建设、循环化改造试点取得实质性进展。

行政改革有力有效。事业单位分类改革、机关事业单位养老保险改革和13家城市公立医院改革稳步实施，城管、工商、政府采购、公共资源交易完成职能并转，5个经济发达镇试点和乡镇统计垂直管理改革有序启动。3次动态调整市县两级权责清单和审批目录，500多项政务服务实现信息共享、简捷办理。“七五”普法扎实推进，出台法治政府建设实施方案，制定首部政府规章[9]，提请人大常委会审议法规草案2件，225件人大代表建议、287件政协委员提案全部办结。完善行政决策五大程序[10]，规范性文件合法性审查试点经验全省推广，依法开展“双随机一公开”监管，预算管理、审计监督、财政资金统筹等一批改革措施精准“滴灌”。

双创活力充分释放。市创业服务中心基本建成，80家企业与高校产学研用合作，新增众创空间25个、院士工作站5家，新建省级创业孵化示范基地1家、研发平台6家。招硕引博48人，接纳实习实训生1.48万人。3家企业获评市第五届质量奖，兑现科技创新劵和创新奖补资金2780万元，提供1.7亿元担保贷款扶持2521人创业，转化重大科技成果92项，发明专利申请量1800件，新登记市场主体 3.41万户，新增高新技术企业38家，培育科技型企业153家。

（四）突出共建共享促和谐，发展成果惠及民生。这一年，财政用于民生的比重达78.6%，资金达283亿元；城乡居民收入增速继续高于经济增速，发展更有温度，幸福更有质感。

脱贫攻坚多向发力。从高设标，建立扶贫工作标准体系和考核对标体系，调整充实驻村帮扶队伍，开展“春季、秋季攻势”和“五项行动”[11]，常态化明察暗访、督导巡查、约谈问责，压实政治责任。整合资金60.6亿元，新建扶贫路175公里，改造农村危房6043户，资助贫困学生12万多人次，医疗救助9万人次，向10058户发放扶贫小额信贷7.36亿元。全市可脱贫84个村56527人，易地扶贫搬迁12425人。

民生保障全面提升。城镇新增就业64322人，失业再就业17576人，城镇登记失业率3.76%。城镇职工养老金13连增，“五项社保” 参保829万人次。向33.82万重点对象发放救助金8.82亿元，改扩建农村福利院15所，安全管理“三个系统”[12]全面建成。在全省中等城市首次举办老博会，高龄津贴提标惠及10万多名80岁以上老人，财政兜底对O～6岁残疾儿童抢救性康复治疗，8万多名残疾人享受“两项补贴”[13]。孝感城区房屋征收户入住城市新社区比例明显提高。棚户区改造19332户，新增租赁补贴发放706户。新增公积金实缴人数1.7万人、资金22亿元。

社会事业协调发展。188所学校“全面改薄”，改扩建幼儿园22所、中小学12所，建成教师周转房505套，宽带网络“校校通”实现全覆盖，进城务工人员随迁子女凭居住证享受义务教育。H7N9疫情防控及时有效。28家市县医院与108家下级医院组建医联体，194个村卫生室改造升级，家庭医生签约服务136.7万人。计生服务向“四个全面”[14]转型，出生人口性别比逐步下降。评选表彰第四届道德模范、第九届“十大孝子”和2名德孝楷模，开展文化惠民活动2000多场次，全国首个膏盐博物馆开馆，成功举办孝文化旅游节、首届澴川读书节、“舞动澴川”龙舟赛和“孝行千里”山地自行车挑战赛，“村村响”“户户通”工程全面建成，竞技体育、文物保护等工作取得新成绩。

平安孝感加快建设。开展孝感城区社区建设试点示范和基层“微平安”创建活动，建立“5+5”治安防控体系[15]，打造“1+5+X”矛盾纠纷多元化解平台[16]，组织开展“四级矛盾联排”，保持对“电诈”“黄赌毒”“盗抢骗”“黑拐枪”和反恐防暴严打高压态势，人民群众“一感两度两率”不断提升。应城六获“全国社会治安综合治理先进集体”，四次蝉联“长安杯”。网上信访成为群众表达诉求的重要渠道，一批信访积案得到化解。安全生产“三个一”机制、“两化”体系、“两个全覆盖”[17]有序推进，“明厨亮灶”“放心早餐店”“双安双创”[18]活动持续开展，安全形势总体平稳。

国防动员、民兵预备役和双拥工作进一步加强，密切与工会、共青团、妇联的联系，人防、保密、气象、水文、档案、史志、物价、供销、保险、慈善、对台、民族、宗教、驻外等各项工作取得了新成绩。

回望2017年，矛盾风险挑战形势严峻，改革发展稳定压力巨大。我们认真贯彻落实中央八项规定精神，坚持“两学一做”学习教育常态化制度化，强化“四个意识”，争做“五个表率”，切实履行“一岗双责”[19]，深入推进政府系统党风廉政建设，反“四风”、改政风、转作风，执行力、落实力不断提升。

在总结成绩的同时，我们也清醒看到，前进路上还有很多困难和问题：一是发展不充分。经济总量、规模以上工业增加值等主要指标没有完成预期目标；没有产值过千亿产业、税收过十亿企业，缺乏大企业、大项目支撑，企业盈利难、成长慢；新登记市场主体同比下降，应对经济下行、保持中高速增长的压力较大。二是发展不均衡。经济质量不高，三次产业结构不合理，传统产业占比高、转型慢，新兴产业仍处于培育阶段，县域经济竞争力不强，社会建设滞后，民生投入不足，区域之间、城乡之间差距拉大，协调发展、共享发展任重道远。三是发展秩序不优。秩序的背后是管理。经济数据逻辑性、匹配性不强，资源环境约束趋紧，市场秩序、政务秩序、城乡秩序不优，标准不一、政出多门、多头管理，离市场主体和人民群众的期盼差距较大。四是发展合力不强。主配角转换不快、眼界不宽、境界不高；部门之间条块分割、推诿扯皮，沟通协调不够；“三高” [20]之下一些干部不愿为、不会为、不敢为，争先进位、创新担当意识不强。对此，我们将不遮掩、不回避、不懈怠，努力补齐短板，全力加以解决。

二、2018年工作安排

2018年，是贯彻党的十九大精神的开局之年，是“十三五”规划中期评估、承上启下的关键一年。孝感正处在逆水行舟、爬坡过坎的关键时期。做好新一年的政府工作，任务艰巨、责任重大。

今年政府工作的总体要求是：全面贯彻落实党的十九大精神，以习近平新时代中国特色社会主义思想为指导，坚持稳中求进工作总基调，坚定践行新发展理念，统筹推进“五位一体”总体布局，协调推进“四个全面”战略布局，扎实推进供给侧结构性改革，着力打好防范化解重大风险、精准脱贫、污染防治攻坚战，推动全市经济发展质量变革、效率变革、动力变革，全面做好稳增长、促改革、调结构、惠民生、防风险各项工作，促进经济发展质量提升、社会和谐稳定，为决胜全面小康社会、建设全省区域性增长极奠定坚实基础。

按照上述要求，我们努力把省委、省政府和市委决策部署具体化、项目化、方案化，立足“两保持一优先”拟定2018年经济社会发展预期目标，即：增速保持全省平均水平，总量保持全省4—6位，坚持质量效益优先，努力争取更好结果，突出抓好“八双”[21]工作，全力推动“六个发展”[22]。

（一）以项目“双增”为支撑，夯实发展底盘。实现“六个发展”，赢得比较优势，就得有物化的项目作支撑、托底盘。我们要广泛开展“三个年”活动，保持“两资一促”主线不断、持续升温，努力实现项目增量增质，扩大有效投资，支撑高质量发展。

开展项目建设提速年活动。梳理100个重点项目，举全市之力提供要素保障、开辟绿色通道、实行“五化”[23]管理，强化分级分类调度、常态化督查、双向激励约束等机制，全力推动项目快开工、早见效，全年实施投资过亿元项目700个以上，固定资产投资增长11%左右。突出抓好市高新区华中光电产业园、孝南维达扩能、汉川好莱客创意家居、云图控股高端品种盐扩能、云梦优尼科光电、安陆天庆彩印、孝昌大洋新动力等亿元以上工业项目建设，工业投资增长12%。力争武西高铁直通线尽早开工，服务好汉十高铁和4条高速路建设，续建新建28条一、二级公路，加快启动孝感至汉川、孝感至应城高速公路，完成交通投资66亿元以上。放宽准入门槛，发挥政府资金引导作用，加快实施一批城乡电网、农田水利、市政工程，推动一批PPP项目落地实施。

开展精准务实招商年活动。强化招商引资“一号工程”，聚焦六大产业[24]，精准对接、精准选商。紧盯武汉市场，瞄准国内外500强区域总部、知名央企和省属企业、现代服务业和新兴产业，招大引强引新，承接产业转移。坚持团队招商、产业招商、校友招商、回归招商、以商引商结合，每个市直招商团队引进1个10亿元以上项目，全市引进亿元项目180个以上，引进内资增长25%，利用外资增长8%，工业项目占70%以上。完善招商项目预审制，建立项目秘书制、履约催办制，持续、动态开展招商引资项目集中签约、开工和拉练活动，力促招商引资向项目落地跟进、全员招商向人人营商转变，市临空区、市双峰山分别开工亿元项目10个和3个，各县（市、区）和市高新区各开工亿元项目25个以上，新签约项目开工率、投产率分别达50%和30%以上。

开展立项争资攻坚年活动。市政府班子成员每人至少争取一个国家或省级改革项目落户孝感、先行先试。推进军工企业混合所有制改革，争取更多军民融合项目布局孝感，以新能源汽车整车资质为牵引，定向争资，精准立项，共圆孝感“汽车梦”。瞄准国家和省重大政策安排，积极申报争取项目、债券和世行贷款，全年争取政策资金和债券150亿元以上。扎实做好第四次全国经济普查。

（二）以城乡“双融”为主导，促进发展转型。从总体看，我国已进入城市和农村资本、资源双向融合的新阶段，产业之间特别是农业领域呈现出一二三产业融合发展的新态势。我们要顺应城乡资本融合、产业融合大势，扶持实体经济和县域经济扩规模、促转型、上档次、壮实力。

推进农旅融合。启动全国农业绿色高产高效整市制创建和全省农业可持续发展试验示范区[25]建设。加快构建现代农业产业体系、生产体系、经营体系，有序引导土地流转，鼓励新型经营主体发展现代农业，农业增加值增长4%，农产品加工业产值增长8%。编制全域旅游、农旅融合规划，围绕“农业+”和“+旅游”，在农业规模化生产中融入旅游元素，发展特色农业、休闲农业、体验农业，鼓励社会资本下乡建设田园综合体，以农促旅、以旅强农。改造升级旅游通道，每个县（市、区）建设一条农旅融合示范线，多点对接武汉旅游线路，打破区域界限，加强资源整合，串点连线、内联外通，面向全省全国打造、推介一批旅游示范线、黄金线。市双峰山创建国家级旅游度假区，全市创建一批特色旅游小镇，培植50个星级农家乐、10个农旅融合示范点、3个湖北旅游名镇名村，力争每年新增1个4A景区，全年接待游客2600万人次，旅游综合收入增长15％。

深化“两化”融合。以信息化促工业化，推进中国制造2025孝感行动，用好用活市级产业发展基金，实施“千企千亿”工程[26]十大技改项目，支持发展智能产业，开展先进制造业试点示范，改造提升三大传统产业，发展壮大三大战略性新兴产业。推进建筑业现代化，适度发展装配式建筑，支持建筑业转型升级。开展质量提升行动，推进品牌强市、质量强市战略，培育一批单项冠军和“隐形冠军” ，争创省级智能制造、服务型制造和“两化”融合示范企业各2家，新增“隐形冠军”6家、驰名商标2件、规模以上工业企业50家以上。

促进区域融合。深化汉孝“五个一体化”[27]，加快建设武大高速，孝汉大道全线通车，陈天大道延伸、硚孝高速二期尽快开工，与武汉开展部门、企业、高校、社团等多层次、多领域交流合作。推进市高新区与武汉国家航天产业基地协作配套，市临空区与武汉临空港错位发展，打通双峰山至木兰景区旅游公路，全方位贯通两地市场和资源。全力支持汉川争创全国县域经济百强县、孝南和应城冲刺全省20强，加快基础设施对接促进孝云一体，支持安陆、大悟、孝昌发展绿色产业，建设鄂北生态屏障。

（三）以动能“双培”为重点，增强发展活力。中国经济正处于新旧动能转换的关键期，我们要抢抓机遇窗口期，坚持培优旧动能、培强新动能，实现新旧动能接续转换、双轮驱动，让发展调速不减势、量增质更优。

深化改革，扩大开放。积极稳妥实施政府职能转变和机构改革，完成县（市、区）城管体制改革。深化农村集体产权制度改革、国有农场改革和医药卫生体制改革。设立市政和资产管理公司和产业投资引导基金，完成市政和担保公司增资扩股、国有资产划转和“三供一业”移交，做强做优湖北澴川国投，力争全年融资授信100亿元。深化供给侧结构性改革，在促转型中去产能，为企业减税降费、降低要素成本10亿元，促进市场主体立恒心增信心。争取设立武汉海关孝感办事处，鼓励童车、皮草、茶叶、麻糖、米酒等特色产品争创省级和国家出口基地，培育外贸服务综合体，引导企业进军“一带一路”市场；加快发展现代服务业，完善县乡村三级物流体系，引进综合电商平台1-3家，新增限额以上商贸单位80家、出口实绩企业40家，外贸出口增长8%。

促进创新，服务创业。启动国家创新型城市创建，稳步推进国家知识产权示范城市建设，建成国家农业科技园区。深化“互联网+”行动，鼓励发展“四新经济”。实施知识产权“五大工程”[28] ，申请发明专利和注册商标各2000件以上，培育和申报知识产权试点示范企业50家、高新技术企业80家，新增省级创新平台10家，高新技术产业增加值增长12%以上。以创业促就业，设立创业导师库、项目库、活动库，开展新型职业农民创业培训，新增省、市创业孵化示范基地2家和6家，加快建设湖北工程技师学院，培养技能人才2000人，城镇新增就业5万人，扶持创业2000人，创业带动就业8000人，困难人员再就业7800人，城镇登记失业率控制在4.5%以内。

繁荣城镇，振兴乡村。实施特色小镇三年行动计划，巩固国家新型城镇化试点成果，全面建成乡镇污水管网和处理设施，完成5个经济发达镇和2个省级特色小镇试点任务，选择50个经济基础好、潜力大的乡镇率先发展，形成新的增长点。按照产业兴旺、生态宜居、乡风文明、治理有效、生活富裕的总要求，推进乡村振兴战略。实施“三乡工程” [29]、农村电商和农业科技工程，开展农村人居环境整治和“厕所革命”三年攻坚行动，加快基础设施、公共服务向农村延伸，实现自然村光纤全覆盖。全域实行“路长制”，加强公路沿线综合管控。建设农村公路600公里，建成59万亩高标准农田和8大水利工程[30]，通过农村垃圾综合治理国家验收，再建100个美丽乡村，新改扩建15所农村福利院，让农村成为扩大投资、拉动消费、市民向往的新天地。

（四）以城市“双修”为载体，筑牢发展平台。在快速城市化过程中，积淀了许多“城市病”，必须以新的理念经营城市。我们要按照国家和省城市“双修”要求，实施城市建设绿色发展三年行动，全面开展生态修复、城市修补，补短板、强功能、优生态、严管理，让城市更美好。

增强城市容纳力。启动孝感市城市总规和云梦、大悟、汉川城市总规修改，编制城市“双修”、地下空间开发等专项规划，努力实现“城市总规一张图、多规合一一枚章、市区联动一盘棋”。着力加强孝感东城新区基础设施建设，完善城市功能，纾解老城区人口和资源压力。推进城市扩容提质，孝感城区完成市政投资100亿元以上。以生态治理、片区开发理念，整合利用棚户区改造、环境治理政策，整体推进老澴河14.68平方公里综合治理，高位推进机场迁建工程，最大限度减少城市“补丁”；动工建设董永北路立交桥，启动交通大道优化改造工程，形成贯通南北、连接东西的城市主轴；拉通、拓宽6条街道[31]，建设2处地下通道[32]，畅通“微循环”；扎实推进国家森林城市创建，新建4大公园[33]，改建7处街头绿地[34]，实施复兴大道东段景观和澴川城市生态绿道工程，统筹建设老城区地下停车场和地面绿化，构建城市绿脉；实施5大供排水工程[35]，加快解决旧城改造遗留问题，综合整治老旧小区，加强物业管理，城区改水6000户，新增天然气用户1.8万户，加快建设静脉产业园[36]、10公里地下综合管廊和15.5平方公里海绵城市。各县（市、区）多方融资，完成城市“双修”投资10亿元以上。巩固深化“城管革命”，构建大城管格局，继续实施“六大秩序”整治，严格推行市县一体城管考核，迎接国家园林城市复查和国家卫生城市省级评估，以创促建促管。

增强园区支撑力。加强各类园区规划统筹，支持各开发区差异化、特色化发展，围绕产业集聚抓配套、企业集中抓服务，加快产城融合，把每个园区打造成产业新城、城市新区。市高新区聚焦“四大四新”[37]，加快“六通一平”配套建设，盘活存量用地，打造全国知名日商产业园，新建电镀产业园，竣工投产20个产业项目，增强综合实力。市临空区完善“三区三园” [38]六大板块配套，推进污水处理厂、天然气工程和凤凰旅游区建设，超前谋划、市区共建临空工业园北部拓展区，迅速形成上项目、聚人气的浓厚氛围。支持各级开发区扩区调区，引导企业集中、产业集聚，推进集群集约发展。

增强环境承载力。完成第二次全国污染源普查和土壤污染详查，开展第三次全国土地调查、水资源调查评价和第五次全省森林资源普查，划定生态保护红线。整改销号中央环保督察反馈的7个问题。建立健全生态环境损害赔偿制度，继续打好大气、水、土壤污染防治三大攻坚战。抓好秸秆禁烧和综合利用、扬尘污染和重点行业深度整治，打赢蓝天保卫战；争取鄂北水资源配置二期工程尽早动工，实施长江大保护九大行动，水岸同治促进河（湖、库）长制常态化落实，提标改造孝感城区污水处理厂，建成邓家河污水处理项目，“一河一策”治理水体污染；坚持“一控两减三基本”[39]，治理农业污染。精准灭荒7万亩，通道绿化500公里，建设绿色示范乡镇2个、绿色示范村100个，朱湖国家湿地公园试点通过验收。严守耕地红线，深化国土资源节约集约示范创建，积极争取新的用地规模，综合运用行政、经济、法律手段，处理好历史遗留问题，提高土地集约利用水平，破解发展瓶颈。

（五）以民生“双保”为追求，共享发展成果。民心是最大的政治，人民满意是我们的最高追求。我们要顺应人民群众对美好生活的向往，坚持人民政府为人民，坚守底线、突出重点、引导预期，主动高效完成上级下达的重点任务，保刚性政策和要求兑现，保民生任务实现，让群众有更多获得感。

扎牢织密社保网络。实施全民参保和社保共享计划，完善城镇职工基本养老保险制度，逐步提高养老金标准。推进第三代社保卡国家试点。合并实施基本医疗保险和生育保险，统一城乡居民基本医疗保险制度，完善异地就医联网直接结算。创新孝老敬老、扶残助弱政策机制，动态提高城乡低保和社会救助标准，落实失能、半失能特困供养人员护理补贴。棚户区改造12911套，建成3404套，新增住房租赁补贴3455户。改造农村危房4000户。新增公积金缴存1万人。加强监管，分类调控，保持房地产市场平稳健康发展，努力实现新建商品房价格涨幅与居民收入增速相适应。

办好人民满意教育。实施学校标准化建设，启动第三期学前教育行动计划，完成“全面改薄”任务，优化孝感城区义务教育布局，适时动工建设东城新区中小学校，化解“大班额”，启动高中阶段教育普及攻坚计划。鼓励发展民办教育， 开展现代学徒制职业教育试点。支持湖北工程学院和湖北职业技术学院加快发展。全面深化新时代教师队伍建设改革，实施“五大能力提升工程”[40]，建立教师岗位目标管理和地方荣誉制度。扎实推进留守儿童关爱保护工程，实施营养餐改善计划，资助各类贫困生12万人次，关心下一代健康成长。

加快建设健康孝感。推进现代医院管理制度试点改革，启动2个市级、建设3个县级卫生项目[41]，每个县（市、区）建一个紧密型医联体，多渠道培养全科医生，家庭医生签约率30%以上，公立医院全面执行药品采购“两票制” [42]，全面实施基本生育免费服务。加快卫生应急体系建设，加强职业病、血吸虫病和重大疾病防控工作。医养结合发展养老产业，培育体育支柱产业，推进2个运动休闲特色小镇试点建设，组队参加省十五运会，公共体育场馆免费向社会开放，开展全民健身活动，以全民健康促全面小康。

持续推进文化惠民。加快现代公共文化服务体系建设，推进各类公共文化设施提档升级。持续开展公益电影放映、文化下乡、全民阅读活动，繁荣发展群众文化和大众文艺，办好省第七届楚剧艺术节、第十三届楚剧展演、第六届民间文艺大赛等文化活动。持续打造“五孝”品牌[43]，大力弘扬社会主义核心价值观。加强公民思想道德建设，完善社会信用体系，广泛开展群众性精神文明创建活动，提升社会文明程度。政府、社会、企业联动，努力把孝感打造成中华孝文化论坛和孝老产业博览会的永久场址，加快建设中华孝文化名城。

（六）以职责“双履”为主线，凝聚发展合力。政府工作必须提高政治站位，做到守土有责、守土负责、守土尽责。我们要在履行好发展主责、分管职责的同时，切实履行好党政同责、一岗双责，促使各项工作齐抓共管、形成合力。

精准推进脱贫攻坚。构建“五方协同”[44]机制，实施“百企帮百村”行动，做实“四大帮扶”[45]品牌，实施片区扶贫攻坚、整村推进、“五个一批”[46]和行业扶贫工程，打好产业扶贫、健康扶贫、金融扶贫、扶志扶智硬仗，突出大悟、孝昌重点，整合资源攻克36个全省深度贫困村堡垒，完成57330名贫困人口脱贫、77个贫困村出列和年度易地扶贫搬迁任务，保证现行标准下的脱贫质量，提升贫困群众认同感、获得感和脱贫荣誉感。

创新推进社会共治。完成村、社区换届选举，建立健全村务监督委员会，加强村务公开、民主管理，推进城乡社区“三社联动”[47]和农村社区建设试点，规范建设一批城乡新社区。加快建设“三个体系”[48]，建立健全社会治理制度和矛盾预防化解机制,推进公共安全视频监控系统联网应用，完成“一标三实”，织密“八张防控网”[49]，开展“十百千”基层平安创建[50]，综合整治“盗抢骗”“黄赌毒”“食药环”等社会突出问题，提升群众的安全感、满意度。加强特殊人群服务管理，抓好社区矫正、戒毒康复、精神病救治和人民调解工作。强化信访属地管理、源头管理、依法治理，积极应对网络舆情，有效预防和化解矛盾纠纷。

扎实推进安全发展。深化金融生态环境建设，发展多层次资本市场，新增“新三板”、四板挂牌企业30家以上，新增贷款速度不低于经济增速、不低于上年增速，有效防范化解企业经营、金融信贷、政府债务风险。开展安全生产打非治违专项行动，加强反恐防暴、道路交通、地质灾害、矿山安全、建筑施工、城镇燃气、社会消防、校园安全、森林防火等专项整治，构建风险隐患双重预防机制，压紧压实安全责任，坚决遏制重特大安全事故。开展优质粮食工程示范创建，严把从农田到餐桌、从实验室到医院每一道防线，建立健全食品药品智慧监管体系，以“四个最严”[51]保障“舌尖上的安全”。建设市减灾备灾中心，强化应急救援演练，提高防灾减灾救灾能力。加强国防教育和后备力量建设，支持国防和军队改革，落实各项优抚政策，扎实创建全国双拥模范城。

（七）以政务“双提”为指向，优化发展环境。良好政务环境就是软实力、竞争力。我们要用敬民之心行简政之道，持续推进政务规范化、信息化和公开化，实现政务服务提速提效。

加强规范化建设。深化“放管服”改革，开展“简政便民”行动，动态调整市级权责清单和审批目录，实行并联办理、容缺审批，推进审批服务简捷化、政务服务标准化，防止以规范化为借口牺牲速度和质效。建成运行全省一流的孝感市民之家，构建覆盖全市、上下联动、功能完善、数据共享、运行高效的政务服务体系，打通服务群众“最后一公里”。落实“双随机一公开”全覆盖，强化事中事后监管，强化行政执法，严厉打击扰乱生产、生活、经营秩序的行为，构建监管有序、竞争公平的市场环境。

加快信息化进程。加强顶层设计,建设全市统一的信息采集、传输、清洗、加工、服务系统，打破信息壁垒，消除信息孤岛，建成并运行市电子政务云计算中心，加快建设智慧孝感。推进政务信息资源全面共享和“云网合一、云数联动”，加快“互联网+政务服务”步伐，完善智慧政务服务平台，开发政务服务APP、开通12345政务热线等操作方便、群众有获得感的终端产品，推行网上受理、网上办理、网上反馈，政务服务事项“应上尽上、全程在线”，促进政务服务线上线下深度融合，实行“最多跑一次”改革，让企业和群众少跑腿、不添堵。

加速公开化步伐。全面推进权力运行公开、政务公开、重大工程建设进度公开，接受社会监督，打造阳光透明政府。依托政府门户网站和政府信息公开联网平台，加强政策解读、回应关切、数据开放，保障公众知情权、参与权、表达权和监督权。把政务“两微一端”纳入政府网站建设，主动、及时、全面、准确发布政务信息。建立市民“随手拍”平台，开辟问政新渠道，提高政策制定、公共管理和公共服务响应速度。

（八）以作风“双严”为标准，强化发展保障。打铁必须自身硬，政府系统自身建设是抓发展的首要前提和根本保证。我们要在市委领导、人大法定监督、政协民主监督之下，以“不忘初心、牢记使命”主题教育为契机，树牢“四个意识”，坚定“四个自信”，力戒形式主义、官僚主义，转观念、转方法、转作风，从严管理、从严要求，增强执行力、公信力，建设人民满意政府。

在政必为。自觉维护党中央权威，坚决把党中央国务院大政方针、省委省政府和市委决策部署落地落实。自觉尊崇法治、敬畏法纪，按法定权限和程序行使权力、履行职责，编制重大行政决策事项清单，遵循重大决策程序，科学决策、民主决策、依法决策；支持司法体制改革，推进综合执法，严格规范公正文明执法，建设法治政府。认真办理人大代表意见、建议和政协委员提案，广泛听取民主党派、群团组织和社会各界意见建议，自觉接受司法监督、舆论监督和社会公众监督，习惯在监督下开展工作。

理政善为。加强学习，以勤补拙，增强“八项本领”，坚持“四种导向”[52]，提高深化改革、推动发展、化解矛盾、维护稳定的能力和水平。强化角色意识，处理好担当主责与当好配角的关系，主动担责，敢于担当。完善正向激励、容错纠错机制，真评实考、追责问责，坚决纠正不作为、慢作为、乱作为问题，为担当者担当，对负责者负责，给干事者鼓劲。

施政实为。自觉在大局中谋划和推动工作，设定目标，高点定位，各县（市、区）和市政府部门单项工作在全省争位次，在全国创特色。紧扣“四向、四言、四子”[53]，践行“一线工作法”，改进作风，沉到一线，在经济社会最前沿发现问题、化解矛盾，在工作推进最难处清除障碍、打开局面。推行政府重点工作“五化”管理，开展执行力大督办，定了干、马上办，抓到底、见成效。

从政廉为。严格落实全面从严治党主体责任，深入推进党风廉政建设和反腐败斗争。严守政治纪律和政治规矩，持之以恒正风肃纪。把廉洁从政贯穿到工作的各个环节，主动接受党内监督、国家监察和审计监督，完善土地出让、工程建设、产权交易、政府采购等制度，加强公共资金、公共资源、国有资产监管，消除权力寻租空间。把廉洁干净作为一条红线、底线和生命线，用制度管权、管事、管人，严肃纠正重点领域不正之风，营造清爽的同志关系、规矩的上下级关系和亲清的政商关系，打造风清气正的公务员队伍。

各位代表，潮起海天阔，扬帆正当时。让我们紧密团结在以习近平同志为核心的党中央周围，在省委、省政府和市委的坚强领导下，高举旗帜，凝心聚力，务实重行，开拓创新，为加快建设全省区域性增长极，全面建成小康社会不懈奋斗！
